# Detection of Nucleic Acid Targets Using Ramified Rolling Circle DNA Amplification: A Single Nucleotide Polymorphism Assay Model

**DOI:** 10.1371/journal.pone.0065053

**Published:** 2013-05-27

**Authors:** James H. Smith, Thomas P. Beals

**Affiliations:** Assay Development Department, Thorne Diagnostics Inc, Beverly, Massachusetts, United States of America; Institut Pasteur, France

## Abstract

**Background:**

Isothermal amplification methods provide alternatives to PCR that may be preferable for some nucleic acid target detection tasks. Among current isothermal target detection methods, ramified rolling circle amplification (RAM) of single-stranded DNA circles that are formed by ligation of linear DNA probes (C-probes or padlock probes) offers a unique target detection system by linked primers and a simple amplification system that is unconstrained by the target’s sequence context. Earlier implementations of RAM-based target detection were reported to be limited by background noise, due in part to unligated C-probe in the amplification reaction. We show here that a target-detection system using a biotinylated target-capture probe together with automated bead-handling reduces or eliminates background amplification noise. We demonstrate the system’s performance by detection of a single-nucleotide polymorphism in human genomic DNA.

**Methodology:**

Target detection by RAM entails hybridization and ligation of a C-probe, followed by amplification and RAM signal detection. We evaluated RAM target detection in genomic DNA using recognition of a human Factor V gene single nucleotide polymorphism (G1691A) as a model. Locus-specific C-probes were annealed and ligated to genomic DNAs that represent the 3 possible genotypes at this locus, then ligated C-probes were amplified by real time RAM. The majority of the steps in the assay were performed with a magnetic bead-based chemistry on an automated platform. We show that the specificity of C-probe ligation permits accurate genotyping of this polymorphism. The assay as described here eliminates some of the background noise previously described for C-probe ligation, RAM amplification assays.

**Conclusion:**

The methods and results presented here show that a combination of C-probe detection, automated sample processing, and isothermal RAM amplification provide a practical approach for detecting DNA targets in complex mixtures.

## Introduction

Several isothermal amplification formats, as well as the polymerase-chain reaction (PCR), have been developed for detection of nucleic acid targets in complex nucleic acid mixtures [1]. Currently commercially available isothermal methods include PCR-like reactions that depend upon multiple enzymes and other components to perform the denaturation/strand displacement function, rather than the thermal cycling of PCR; these include helicase dependent amplification (HAD) [2] and recombinase polymerase amplification (RPA) [3]. Like traditional PCR, these methods require a target-specific primer pair. In contrast, isothermal loop-mediated amplification (LAMP) [4] requires multiple sets of primers that flank the target locus.

**Figure 1:**
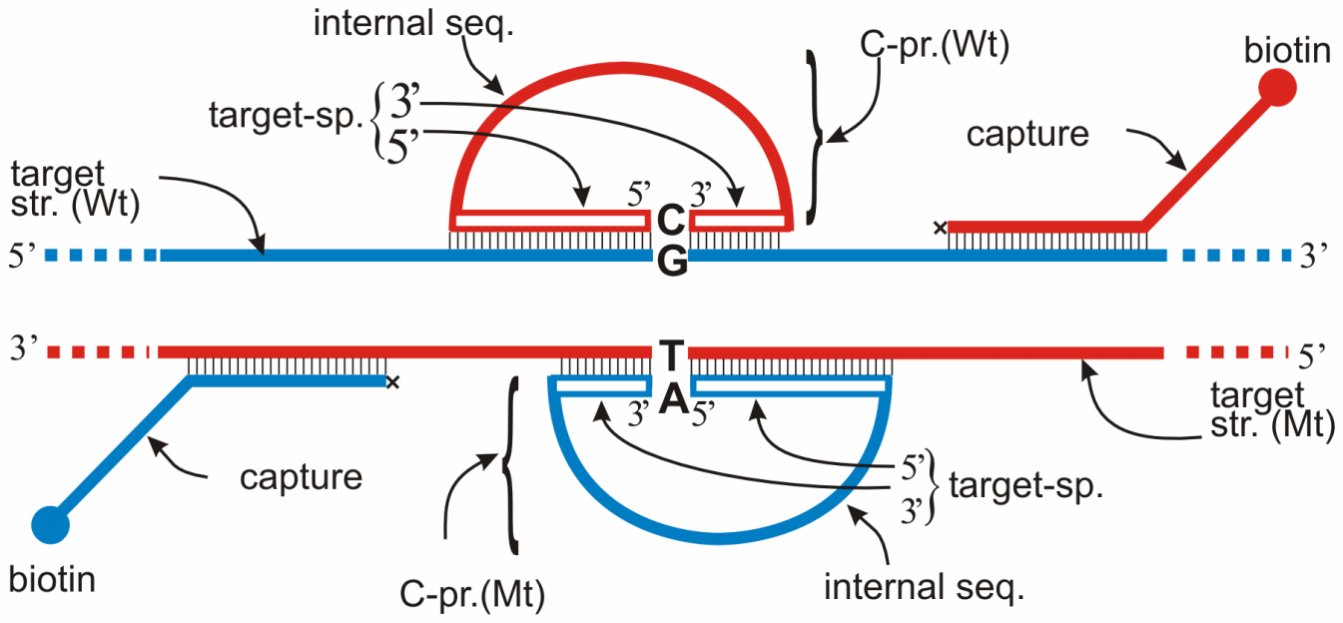
Nucleic acid species used for detection of alleles at a locus via the RAM reaction. The figure shows a wild type (Wt) target strand in blue (“target str. (Wt)”) and a mutant (Mt) target strand in red (“target str. (Mt)”). A C-probe (“C-pr. (Mt)” and “C-Pr. (Wt)”) is shown bound to each target strand via target-complementary 5′ and 3′ target-specific segments; the C-probe’s internal sequence (“internal seq”) is not complementary to the target sequence. The SNP base is shown in bold-face in the target strands and in the C-probes. A 3′ blocked (x) capture-probe that terminates in a 5′ covalently bound biotin molecule is drawn annealed to each DNA strand.

RAM reactions require a single stranded circular DNA, a single enzyme and a primer pair. Following target recognition and linear C-probe ligation, the circularized single-stranded DNA (ssDNA) C-probe (Figure 1) is amplified, rather than the target sequences. Amplification primers can be directed at a non-target sequence in the body of the C-Probe (“internal seq” Figure 1). Separation of target recognition and amplification sequences allows flexibility in primer design, because choice of amplification primers is unconstrained by the target context.

Ligation of linear ssDNA probes to form ssDNA circles has been used in a variety of assay formats. Originally designated as padlock probes [5], linear molecules with gene-specific termini separated by a spacer have also been called C-probes [6], open circle probes [7], or molecular inversion probes [8]. Initially implemented as simple ligation-closure tools, circles can also be closed after template-directed filling of a gap between the gene-specific termini [9]. ssDNA circle amplification can be accomplished via ramified DNA amplification using a pair of primers [6], by rolling circle amplification initiated by a single primer [9], or by PCR [8]. The RAM amplification can be implemented in either a real time quantitative [10], [11] or endpoint format [12].

Earlier implementations of RAM were reported to be noisy [7], [13], but here we describe conditions that allow reliable, low-noise RAM reactions. We illustrate those conditions with reagents that detect the G1691A SNP in the human Factor V gene [14] that encodes a clotting cascade component. Our objective here is to show that C-probe ligation, RAM amplification assays utilizing substantial automation can be done at acceptable signal-to-noise levels. We also describe briefly some C-probe and RAM primer design methods. We show that these reagents detect the SNP in heterozygotes and in both homozygous forms, and we describe C-probe and primer selection rationales. These methods were implemented in a large scale comparison of RAM-based assays to FDA-cleared assays in collaboration with a clinical laboratory (manuscript in preparation).

## Materials and Methods

**Figure 2:**
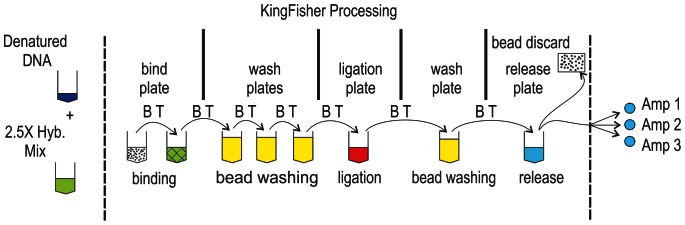
Sample processing sequence for a RAM assay. The figure illustrates the assay process for a single well on a KingFisher 96 well plate. The steps between the vertical dashed lines are performed as an automated sequence. Arrows indicate sequential bead transfer (BT). Beads are removed in the final step prior to RAM.

Figure 1 depicts conceptually the structure of a pair of C-probes that detect a SNP on wild-type and mutant DNA strands, as well as a pair of strand-specific biotin-labeled capture probes that allow the ternary (capture/C-probe/target) complex to be bound to a streptavidin-coated bead. Figure 2 shows an overview of this RAM-assay-based process including both automated and manual steps. DNA samples were fragmented, and then incubated under DNA hybridization conditions with SNP-specific C-probes and biotin-tagged capture probes. After hybridization, the binding of the ternary complex to streptavidin-coupled magnetic beads, bead-washing, C-probe ligation, and sample suspension in a RAM-assay-ready form followed as automated steps. Real-time signals were recorded for each RAM reaction via SYBR-Green fluorescence monitoring.

### C-probes

C-probes with gene-specific termini were designed with longer 5′ (Figure 1, “5′ target sp.”) and shorter 3′ (Figure 1, “3′ target-sp.”) regions. 5′ gene-specific regions were designed with predicted melting temperatures that were in the range of 12° – 15°C greater than the hybridization temperature. The 3′ nucleotide of the linear C-probe is specific to the variant SNP base (Figure 1) [15]; the final length of the target-complementary 3′ gene-specific sequences were adjusted to optimize SNP discrimination in the assay. The C-probe design rationale is that initial and stable 5′ end binding restricts diffusion of the C-probe’s shorter 3′ gene-specific segment to the target region, thereby increasing the 3′ end’s effective local concentration.

The minimum length that is required [16] for C-probe function is greater than the sum of the terminal target-specific sequences used here; an internal sequence (Figure 1) separates the target-specific sequences. The internal sequence may contain functional modules such as hybridization tags or primer-binding sites. Candidate C-probe internal (non-gene-specific) sequences (Figure 1, “internal seq”) were evaluated using rule-based and empirically derived criteria; including, for example, elimination of internal structures such as stem-loops that would be stable under hybridization and assay conditions.

### Primers Selection

The C-probe design-phase included selection of an amplification primer pair from a candidate set that was initially selected using Primer3 [17]. Lack of signal in primer-only RAM reactions was required for provisional acceptance of any given primer pair. Primer pairs were tested in real-time RAM assays with preformed circularized C-probes as templates; performance characterization included testing various template levels with different primer concentrations.

### Capture Probes

Capture probes provided an additional measure of specificity by annealing to a defined sequence flanking the SNP locus of interest; the 5′-linked biotin (Figure 1, “Capture”) allowed the capture-probe to be bound by a magnetic-bead-coupled streptavidin moiety. (Capture probes annealed to the plus and minus target DNA strands are shown in Figure 1.) Alternatively, a single capture probe and SNP-specific C-probes that have the same gene-specific sequences (except for the 3′ terminal SNP base) are also effective in the assay as described (data not shown).

### Samples Analyzed

**Figure 3:**
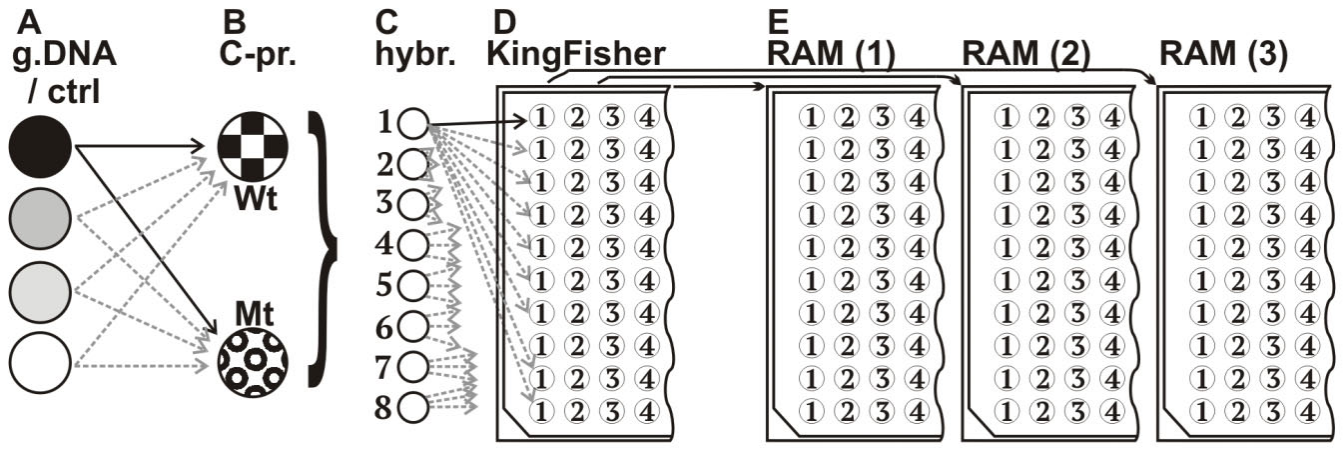
Experimental design for SNP assay. The figure represents a procedure that was followed on each of three days. (A) Three genotypes of genomic DNA or no-DNA control were combined with (B) each of two C-probes in (C) eight hybridization tubes. After hybridization, ten aliquots from each hybridization reaction were transferred to a Kingfisher plate (D). Following the automated process as described in the text, aliquots from each Kingfisher well were transferred into (E) three RAM reaction plates. Some arrows between steps A and B are in light gray for visual clarity. Each hybridization tube (C) was aliquoted into 10 wells in the KF plate (D); some arrows between (C) and (D) are shown shorter for visual clarity. The plate layouts are illustrated for conceptual clarity and do not correspond to physical plates.

On each of three days, an aliquot of each fragmented denatured DNA or no-DNA control was hybridized independently to both C-Probes (and corresponding capture probes) to give a total of 8 hybridization tubes (Figure 3) that were processed as described below. Post-hybridization, 10 aliquots (55 µl) from each tube were transferred to 10 wells of a 96 well KingFisher bind plate, filling 80 wells. Figure 3 shows a diagram of the analysis of a single genotype/probe combination. At the conclusion of a KingFisher run, 3 aliquots per well in the resulting release plate were amplified in 3 RAM amplifications. This process generated 30 results for each DNA or no DNA probe combination per run, and 90 results for each combination over the 3 days.

### Genomic DNA Preparation

For these collaborative experiments that simulate clinical laboratory sample preparations, restriction endonuclease digestion was used to prepare genomic DNA for analysis, although this method would not necessarily be chosen for a RAM-assay-optimized work flow. Genomic DNA samples NA0536 (Factor V Wt), NA16889 (Factor FV Mt), and NA16028 (Factor V heterozygote) were obtained from the Coriell Institute for Medical Research (Camden, NJ). Based on the vendor’s specifications and prior to endonuclease digestion, the DNAs were diluted to 6.7e3 genomes/µl. 320 µl of each type of diluted DNA was digested in a final volume of 1600 µl. Digestion was performed in 1X New England Biolabs (NEB, Ipswich, MA) restriction-enzyme-buffer 4 containing 30 units/ml of BsaI, FokI and HaeIII (NEB) at 37°C for 1 hour, generating 576 nucleotide target DNA fragments. A mock digest was included for the no-DNA control. Immediately before hybridization the fragmented samples were denatured for 10 minutes at 95°C.

### Hybridization

250 µl of Wt or Mt FV C-probes in 2.5X hybridization solution were separately combined with 375 µl of each genotype from a fragmented DNA master digest (1.33e3 genomes/µl) or with a no-genomic-DNA control. Samples were held for hybridization at 52°C for 1hour. 55 µl aliquots from each hybridization tube were distributed into 10 individual wells of a 96 well KingFisher plate (Figures 2 and 3).

### Kingfisher

The following steps, shown in Figure 2, were carried out on a Kingfisher 96 (KF) automated sample-processing platform (Thermo Fisher Scientific Inc.) that was equipped with a disposable-sleeve-covered magnetic probe. The KingFisher instrument transfers magnetic particles to a succession of 96 well plates.

55 µl of each hybridization tube was added per well to a KF 96-well plate. That plate (bind plate, Figure 2) was transferred to the KF deck, with a bead plate (50 µl/well), 4 wash plates (100 µl/well), a ligase plate (50 µl/well) and an elution plate (50 µl/well). In an automated sequence (Figure 2), beads were added to the hybridization mixture and held for 10 minutes at room temperature to allow capture-probe binding onto magnetic beads. Beads were washed three times by transfer to 3 separate wash plates, then transferred to a plate containing ligation buffer and held at 52°C for 2 minutes. Following post-ligation wash, beads were dispensed into an elution plate containing low-ionic strength elution buffer and RAM primers, under conditions that release the circularized C-probes from the magnetic beads. In the final automated step the beads were removed, leaving an amplification substrate.

### RAM

For amplification 10 µl of bead-eluate was combined with 10 µl RAM reaction mix (Figure 2). For the process described here, 3 sequential amplification reactions were performed from each well in 3 RAM plates. Isothermal RAM reactions were performed at 63°C for 90 minutes in an iCycler (Bio-Rad, Hercules, CA) real-time fluorescence reader. Under these conditions the cycle threshold (Ct) reported by default settings of the iCycler iQ version 3.1 software is interpreted as a response time (Rt; [10], [18]). A 40 minute Rt acceptance-limit for positive samples was used following Poisson failure analysis (unpublished data) of RAM reactions on dilution series of preformed circle templates.

### Materials

All C-probes, capture probes and RAM primers were synthesized by Gene Link Inc., Hawthorne, NY. Both C-Probes and capture probes were gel- purified. The target-specific capture-probe sequences used were: CpFctV+: (5′ TCAGAATTTCTGAAAGGTTACTTC), and CpFctV–: (5′CCTCTGGGCTAATAGGACTACTTCTAATCTG) and both capture probes were modified with a 5′ biotin moiety and with a 3′ spacer C3 moiety (Figure 1, “x”) to block 3′ extension by the DNA polymerase.

The C-probes used were: Cpr8FVWt1: (5′GCCTGTCCAGGGATCTGCTCTTACAATACGAGAACACCCGATTGAGAGAGTTTGGAAGTGTAGGCGTGAAGTCCATAACACATACCTGTATTCCTC), and Cpr9FVLdn1: (5′AGGAATACAGGTATTTTGTCCTTGAAGTAACCCTCGTGAAAGCCCTACTCTATGAAATCTTGTAGCAGGACTCCGTTTAGCAGCACTGGACAGGCA). Their gene-specific termini target Factor V Wt and Mt alleles respectively, as illustrated in Figure 1.

C-probes were kinased in 1X Kinase Buffer, 1 mM rATP, 200 U/ml T4 Polynucleotide Kinase (NEB, Ipswich, MA) and 6 µM C-probe. Incubation for 30 minutes at 37°C was followed by enzyme deactivation at 65°C for 30 minutes.

2.5X Hybridization probe mixes contained: 25 mM TrisHCl pH 7.9, 2.5 mM EDTA, 1.5 M NaCl, 0.25% Triton X-100 in addition to 2.5 nM capture probes (CpFctV+ or CpFctV-) and 0.4 nM Cpr8FVWt1 or 0.25 nM Cpr9FVLdn1.

SeraMag Streptavidin Particles (Thermo Scientific, Indianapolis, IN); part No. 3015105010150; biotin-binding capacity 4559 pmole biotin/mg particles; suspension (0.025% solids) were prepared by 2×20 minute washes in 400 µl 1X BlockIt (ArrayIt Corporation, Sunnyvale, CA). 50 µl of beads resuspended to 0.025% solids in 1X hybridization buffer was added per well of a bead source KF plate.

Wash buffer: 10 mM TrisHCl, pH 7.9, 1 mM EDTA, 0.1% Triton X-100.

Ligation mix: 1X NEB DNA Ligase Buffer, 10 Units/ml Taq DNA Ligase (NEB).

Elution buffers: 2.5 mM TrisHCl, pH 7.9, 0.25 mM EDTA, 0.1% Triton X-100, and 2.5 µM Cpr8FVFwd61_22 (ACACTTCCAAACTCTCTCAATC) and 2.2 µM Cpr8Rvs03_20(CTGTCCAGGGATCTGCTCTT) or 2.5 µM Cpr9Fwd73_21 (GAGTCCTGCTACAAGATTTCA) and 1.5 µM Cpr9Rvs87_21 (TGGACAGGCAAGGAATACAGG).

RAM amplification buffer: 45 mM Tris Acetate pH 8.3, 80 mM potassium acetate, 2 mM MgCl_2_, 0.1% Triton X-100, 0.001% Antifoam SE-15 (Sigma, St Louis, MO, catalog number A8582), 0.13 U/µl Bst DNA Polymerase, large fragment (NEB), 200 µM dNTPs, 0.12X SYBR-Green (Molecular Probes, supplied as 10,000X stock), 1 nM fluorescein isothiocyanate (FITC, Bio-Rad, Hercules, CA), 10% dimethylsulfoxide (DMSO).

### Statistical Analysis

Rt data with experiment day, hybridization tube, Kingfisher plate and RAM plate were imported as a data-frame into the “R” statistical environment [19]. Non-parametric tests of Rt data for C-probe/target comparisons within and among plates, days, and of Rts for Wt vs. Ht or Ht vs. Mt, were done by Kruskal-Wallis rank-sum tests supplied in the R ‘stats’ package. P values below the 0.05 level were considered significant.

## Results and Discussion

The assay described here was developed, optimized and evaluated as a prelude to testing patient samples derived from whole blood, purified by the MagnaPure System (Roche). The level of targets tested is within the range of target levels expected in such samples [20]. The goal of the optimization was to achieve high specificity, by varying probe concentration, hybridization time, ligation time and temperature.

**Figure 4:**
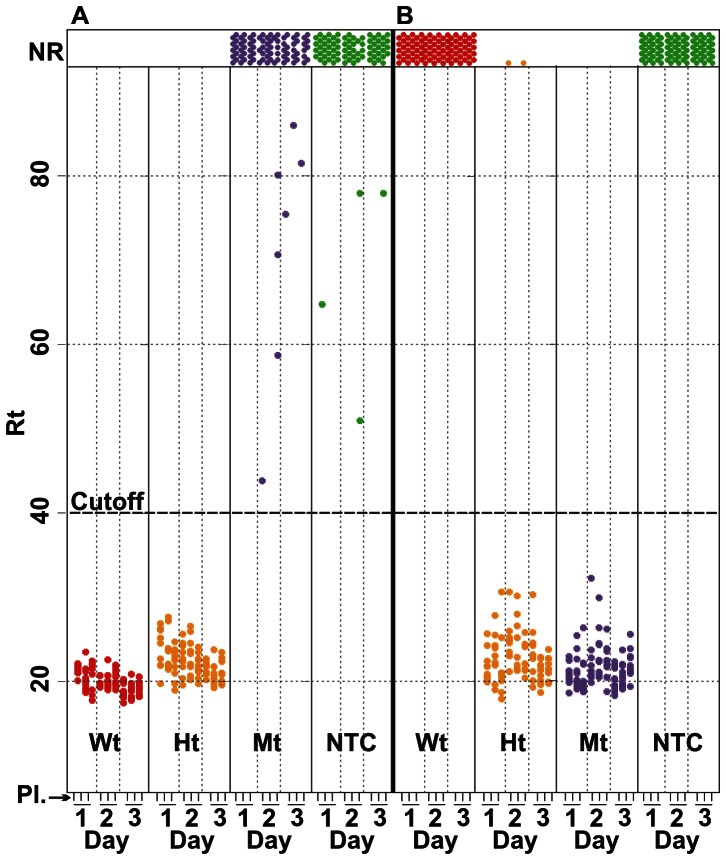
RAM assay data from C-probes ligated on genomic DNA targets. Panels A and B show the RAM response times (Rt) for DNA samples hybridized to the Wt- and Mt-detecting C-probes, respectively. Columns within panels are labeled with the target DNA genotype or with “NTC” (no-target control). Rt signals from nine RAM plates (“Pl”; see Figure 3) that were run on each of three days (Day) are shown vertically in columns. Each NR result is shown in the top panel (NR).

Three Factor V genotypes and one no-DNA control were combined with two (Wt and Mt) SNP-detecting C-probes, resulting in eight combinations of C-probe type plus genomic DNA or no-DNA control (Figure 3). Figure 4 shows response times for the eight types of DNA/probe combination samples. The probe and DNA target combinations are represented within columns. The vertical axis represents the response times for amplification-positive samples. The amplification-negative, or no-response (NR) samples, are defined as wells with no fluorescence increase after 90 minutes amplification and are shown in a “NR” panel.

A qualitative analysis, scoring Rt results as positive or negative is shown in Tables 1 and 2. The Wt-detecting C-probe (Table 1) detected the Wt and Ht DNA in all replicates (90/90 for both types of DNA samples). The Wt probe yielded mostly NR wells, (83/90) and (86/90) respectively with the Mt genomic DNA and no-DNA samples, but also generated late (>40 minutes) signals that do not obscure experimental results. These non-NR results are infrequently encountered where the expected result is NR.

**Table 1:** Qualitative analysis of the results from the genomic DNAs vs Wt probes.

DNA	Rt < = 40	Rt>40	NR	Sensitivity	Specificity
Wt	90	0	0	100	
Het	90	0	0	100	
Mut	0	7	83		100
None	0	4	86		100

Wt probe analysis

**Table 2:** Qualitative analysis of the results from the genomic DNAs vs Mt probes.

DNA	Rt < = 40	Rt>40	NR	Sensitivity	Specificity
Wt	0	0	90		100
Het	88	0	2	98	
Mut	90	0	0	100	
None	0	0	90		100

Mt probe analysis.

The 4 non-NR results observed with the Wt C-probe and the no-DNA samples (Figure 4, panel A; column “no-DNA”) may be due to non-circularized C-probe in the amplification. The 7 fluorescence-positive results with Rts greater than 40 minutes observed with the Wt C-probe and Mt DNA (Figure 4, panel “A”; column “Mt”) may represent amplification of low-level mismatched ligation of a fraction of the C-probes (discussed below) and/or linear C-probe, as for the no-DNA sample. In previous experiments we have encountered intermittent infrequent late Rts with the Wt probe in negative samples. This suggest there are factors involved in the assay which are not as-yet understood, but do not impair interpretation of the results. The total 11 late positive results arose from 10 individual bead eluate wells. One well in this category produced 2 out of 3 positive results (Rts >80 minutes) and the other 9 were from different wells. In general this noise does not correlate with hybridization tube, KF well, or RAM plate.

Mt-detecting C-probes (Table 2) hybridized to the Mt genomic DNA sample yielded all-positive amplification results, but the results from the Ht DNA yielded 88 positives and 2 negative results (Figure 4, panel B, column “Ht”). The negative results came from a single KF well whose third amplification result was positive (Rt  =  21.4 minutes). This Rt value is within the range of the other positive results in this category. We believe that the simplest explanation for this inconsistency is a manual liquid handling error. The Wt DNA and the No DNA samples hybridized to Mt probes were completely negative (Figure 4, panel B, columns “Wt” and “no-DNA”). It is instructive that the (expected) negative results with the Mt probe are all NR, whereas the anticipated negative results with the Wt probe hybridized to Mt DNA and no-DNA produced 11 late positives, indicating C-probe specific noise.

In summary, with a 40 minute cutoff (Tables 1 and 2), the overall specificity of the SNP assay for both probe sets was 100% as there were no false positives; the sensitivity of the Wt probe for both Wt and heterozygote samples was 100%. One anomaly arose with the Mt probe set. The sensitivity of this probe annealed to Mt DNA was 100%, however with heterozygous DNA samples the sensitivity was 98% in this study.

Statistical analysis was done at several levels. Figure 4 shows that the 40 minute cutoff for positive signals is flanked, here, by an approximately 10 minute, no-signal window. The non-overlapping distribution of signal-data-points vs. noise makes first-pass data analysis relatively straightforward.

**Figure 5:**
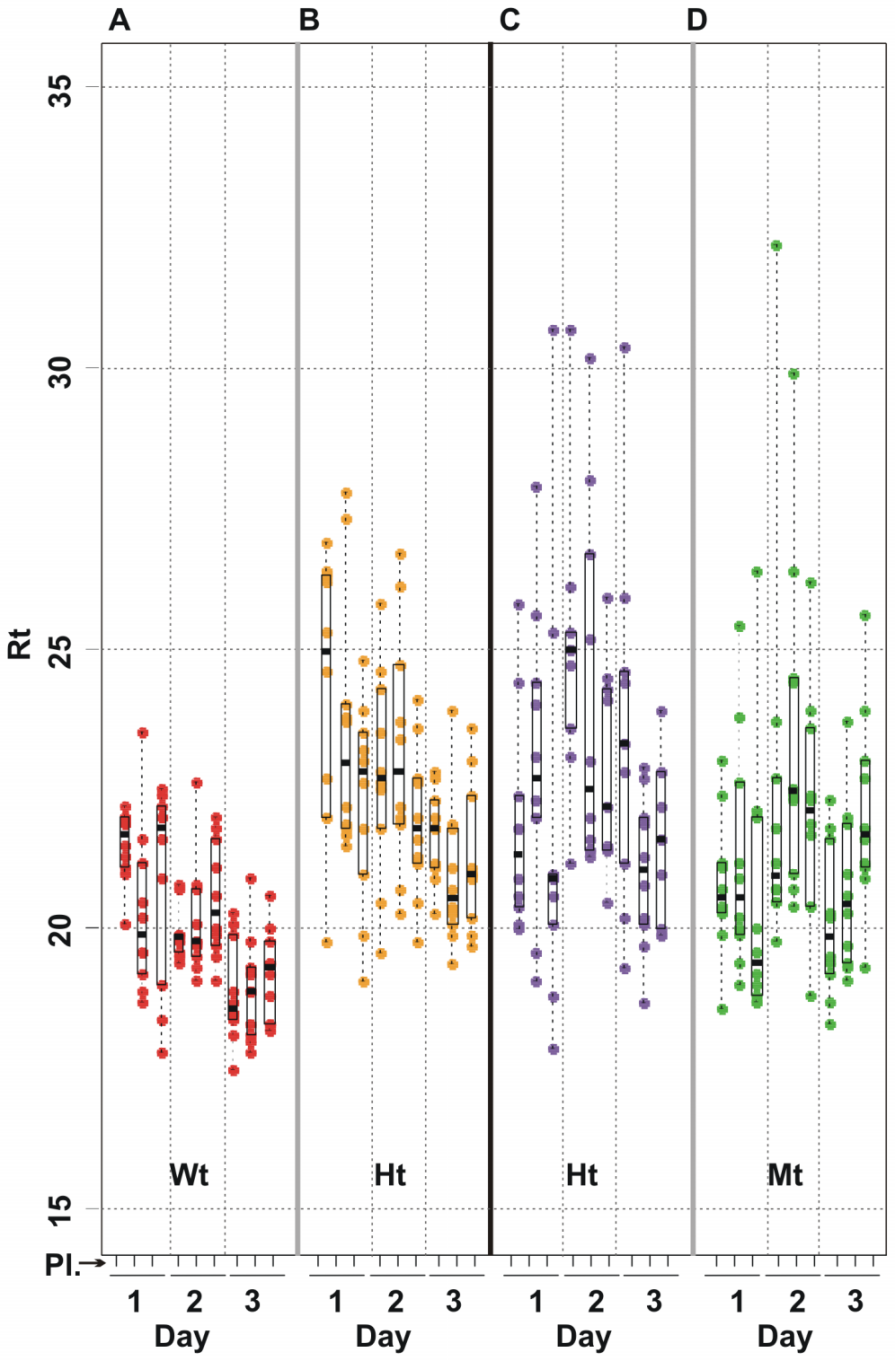
Detail view of homozygote vs. heterozygote RAM response times. Panels A and B show the RAM response times (Rt) for Wt and heterozygote DNA samples hybridized to the Wt-detecting C-probe. Panels C and D show the RAM response times for Ht and Mt DNA samples hybridized to the Mt-detecting C-probe, respectively. The data are marked with interquartile graphic boxes; dotted lines extend to maximum and minimum signals and a bar marks the Rt median value.

More detailed analysis is instructive for the process, although not required for the primary goal. Tables 3 and 4 show that, for each of the C-probe, target combinations shown on Figure 5, a non-parametric assessment of Rt within plate and day shows that, except for one plate, plates are comparable within days. That is, we cannot reject the null hypothesis that Rts are similarly distributed among plates within days. Table 4 shows that days are distinguishable when Rts are pooled within days, then tested for similarity between days. This statistical significance does not appear to us to have interpretive significance.

**Table 3:** Comparison of Rts from plates within days.

C-probe	target	day	p-value
Wt	Wt	1	0.1618
Wt	Wt	2	0.4525
Wt	Wt	3	0.6443
Wt	Ht	1	0.2099
Wt	Ht	2	0.3716
Wt	Ht	3	0.2044
Mt	Ht	1	0.2788
Mt	Ht	2	0.1476
Mt	Ht	3	0.1124
Mt	Mt	1	0.3042
Mt	Mt	2	0.3535
Mt	Mt	3	0.0462

“Are plates different within a day?” A non-parametric test of the null hypothesis of indistinguishable C-probe/target combinations from plates within days, provides a per-plate p-value. Graphically, individual verticals representing plates within day (Figure 5) are being compared.

**Table 4:** Comparison of Rts among days.

C-probe	Target	p-value
Wt	Wt	3.0E-07
Wt	Ht	2.8E-04
Mt	Ht	2.0E-03
Mt	Mt	3.7E-03

“Are days different?” A non-parametric test of the null hypothesis of indistinguishable days for C-probe/target combinations between days provides a per- C-probe/target combination p-value. Graphically, Rts combined from plates within days (Figure 5) are being compared between days.

However, Table 5 suggests statistical support for significance of the apparently distinct Rts of Wt C-probes compared between Wt vs. Ht genotypes (Figure 5A and 5B) when tested platewise (that is, we compared Rts for wells on the same plate). Although our design goal was a qualitative (yes/no) assay it is possible that these Rt differences are due to target copy number in the heterozygous vs. the homozygous genotypes. While significant differences are seen only for two out of nine plates for the Mt C-probe compared between Ht and Mt genotypes, it is possible that outlier removal might reveal plausible differences. We do not pursue that possibility further.

**Table 5:** Within plate comparison of Rts between C-probe-homozygote and C-probe-heterozygote.

C-probe	Comparison	Plate	Day	p-value
Wt	WtRt v HtRt	1	1	0.0063
Wt	WtRt v HtRt	1	2	0.0022
Wt	WtRt v HtRt	1	3	0.0002
Wt	WtRt v HtRt	2	1	0.0009
Wt	WtRt v HtRt	2	2	0.0017
Wt	WtRt v HtRt	2	3	0.0011
Wt	WtRt v HtRt	3	1	0.0587
Wt	WtRt v HtRt	3	2	0.0283
Wt	WtRt v HtRt	3	3	0.0009
Mt	HtRt v MtRt	1	1	0.2892
Mt	HtRt v MtRt	1	2	0.0072
Mt	HtRt v MtRt	1	3	0.0127
Mt	HtRt v MtRt	2	1	0.1304
Mt	HtRt v MtRt	2	2	0.5966
Mt	HtRt v MtRt	2	3	0.5201
Mt	HtRt v MtRt	3	1	0.3068
Mt	HtRt v MtRt	3	2	0.5133
Mt	HtRt v MtRt	3	3	0.5697

“Are Rts for homozygotes vs. heterozygotes different?” A non-parametric test of the null hypothesis of indistinguishable Rts between homozygote and heterozygote provides a per-plate p-value for each of two C-probes. Graphically, verticals representing Rts from corresponding plates (Figure 5, panels A, B and C, D) are being compared.

Several crucial differences distinguish the assay as described above from earlier implementations of a two-primer ramified rolling circle assay used to detect SNPs. Our method of real-time monitoring of the RAM reaction uses SYBR-Green as a fluorescent indicator, in contrast to Faruqi [7] who also hybridized linear C-probes (called open circle probes (OCP) by them) directly to unamplified genomic DNA, but used a quencher- and fluor-containing primer for product detection. Other differences include our use of a capture probe, and the substantial automation of our procedure. Pickering [21] and Ghouze [22] revised the methods described by Faruqi et. al. The two latter groups each amplify SNP loci by PCR prior to annealing the OCP, adding potential amplification bias and substantial overhead to the workflow. The latter two groups, as well as Faruqi et. al., discuss the problem of background signal noise due to the presence of unligated OCP (see below).

The use of a biotinylated target-capture oligonucleotide in the assay allowed several simultaneous optimizations. First, recognition of the target by the capture probe provides an additional measure of selectivity and can serve as a cleanup step to retrieve the target strand from a crude cell lysate, isolating the capture-probe, C-probe, target complex away from potentially enzyme-inhibiting components, which can negatively affect ligation and/or amplification reactions [23].

There are at least three distinguishable noise sources in RAM assays. RAM primers alone in RAM amplification can give rise to detectable double-stranded DNA product. This primer noise signal, like primer-only noise in PCR, can be eliminated by judicious choice of RAM primers (unpublished data). Linear C-probe can result in a noise-signal in RAM amplification [7], [13] through an as-yet unknown mechanism. Hafner et. al.[13] described a ligase-independent amplification/multimerization reaction with primers directed towards a short target region in genomic DNA – a PCR like reaction in the absence of thermocycling. In the same publication, to increase the specificity of their cascade rolling circle amplification (RAM), they recommend chromatographic removal of non-ligated C-probe prior to amplification to increase the specificity of their assay. Faruqi et. al. [7] mention that their unligated OCPs can act as both templates and primers giving rise to non-specific DNA synthesis. They recommend designing hairpin-like structures on the 3′ end of the C-probe, which should render the unligated probes double stranded during the amplification. In our process the post hybridization capture and washing of target probe complexes from the sample prior to ligation and amplification reduces the carry-over of linear C-probe. Both primer-only and linear C-probe reaction noise products can be distinguished from closed-circle derived RAM reaction products by gel or capillary electrophoresis, by analysis of their real-time signal [10], [11], or sometimes by product DNA melting temperature profile (unpublished data).

A third source of noise in RAM reactions is ligation of a C-probe on a non-homologous SNP target. Those reactions are rare [7], [24] but can occur, resulting in ssDNA circles that are identical to circles formed after ligation on complementary template DNA. The assay as described seeks to minimize those reactions by optimizing the gene-specific ends of the C-probe, and by varying ligation reaction time and conditions. RAM products generated from C-probes ligated on a non-homologous target are indistinguishable from specific ligation RAM products by the methods mentioned earlier. However, as a rare event, those ssDNA circles are expected to produce Rts much later than Rts from a homologous ligation. The late Rt results seen in our data with the Wt-detecting probes and the Mt DNA could be explained by either linear C-probe noise or non-homologous probe ligation noise, or both, whereas the no-DNA sample results may be due to noise from linear C-probes. The lack of noise in 360 RAM assays where negative results were expected (in 180 DNA containing and 180 no-DNA controls) makes post-reaction signal analysis relatively straightforward. Late response times can be excluded by imposing a response time cutoff value.

Assays for SNP discrimination are a stringent challenge for a nucleic acid target detection test. Other types of target detection have less rigorous demands. There are scenarios where closely related targets are unlikely to be present, e.g. bacterial or fungal targets in blood, or in differentiating microbial species where related targets can differ at several locations (base substitutions, deletions or insertions), e.g. Mycobacterium [25], Chlamydia [26], or fungi [27]–[29]. Our SNP assay performance suggests that the RAM assay, capture probe, automated platform and RAM detection system may have utility in these situations. The process described here is well-suited for moderate sample throughput, and additional automation is possible. The option of a primer pair unrelated to target sequences may be of advantage in some situations over PCR and other isothermal amplification approaches.

The current automation was developed from an earlier tube-based assay format, similar to that described by Zhang et. al. [6] where manual bead separation involved partitioning the magnetic beads on the wall of the tubes and liquids were discarded, or by bead suspension transfer by aspiration to fresh tubes. In contrast as described above, the KingFisher instrument transfers beads platewise among wells. The manual format is useful for initial assay development where novel analytes are being detected, and is practical for analyzing small numbers of samples.

Multiplex detection of targets should be possible as the internal portion of the C-probe may also be designed with tag domains to facilitate assay multiplexing (unpublished results). As performed here, the fluorescent dye detection during amplification is generic with no need for target-specific fluor-coupled oligonucleotides. We conclude that the isothermal RAM reaction and capture probe/C-probe assay specifically detects ligated C-probe circles and provides an efficient workflow on an automated platform.
